# Sirtuin 1 and Vascular Function in Healthy Women and Men: A Randomized Clinical Trial Comparing the Effects of Energy Restriction and Resveratrol

**DOI:** 10.3390/nu15132949

**Published:** 2023-06-29

**Authors:** Gustavo Henrique Ferreira Gonçalinho, Karen Lika Kuwabara, Nathalia Ferreira de Oliveira Faria, Marisa Fernandes da Silva Goes, Alessandra Roggerio, Solange Desirée Avakian, Célia Maria Cassaro Strunz, Antonio de Padua Mansur

**Affiliations:** 1 Faculdade de Medicina, Universidade de São Paulo, São Paulo 05508-060, Brazil; gustavo.goncalinho@usp.br (G.H.F.G.); karenkuwabara@gmail.com (K.L.K.); nathaliafariaof@gmail.com (N.F.d.O.F.); 2 Serviço de Prevenção, Cardiopatia da Mulher e Reabilitação Cardiovascular, Instituto do Coração do Hospital das Clínicas da Faculdade de Medicina da Universidade de São Paulo (InCor-HCFMUSP), São Paulo 05403-900, Brazil; 3 Pesquisa Clínica, Instituto do Coração do Hospital das Clínicas da Faculdade de Medicina da Universidade de São Paulo (InCor-HCFMUSP), São Paulo 05403-900, Brazil; marisa.goes@incor.usp.br; 4 Laboratório de Análises Clínicas, Instituto do Coração do Hospital das Clínicas da Faculdade de Medicina da Universidade de São Paulo (InCor-HCFMUSP), São Paulo 05403-900, Brazil; alessandra.roggerio@incor.usp.br (A.R.); labcelia@incor.usp.br (C.M.C.S.); 5 Unidade Clínica de Cardiopatias Valvares, Instituto do Coração do Hospital das Clínicas da Faculdade de Medicina da Universidade de São Paulo (InCor-HCFMUSP), São Paulo 05403-900, Brazil; solange.avakian@incor.usp.br

**Keywords:** sirtuin, resveratrol, calorie restriction, endothelial function, vascular function, noradrenaline, sympathetic nervous system, vascular reactivity

## Abstract

*Background*: Sirtuin 1 (SIRT1) has been associated with longevity and protection against cardiometabolic diseases, but little is known about how it influences human vascular function. Therefore, this study evaluated the effects of SIRT1 activation by resveratrol and energy restriction on vascular reactivity in adults. *Methods*: A randomized trial allocated 48 healthy adults (24 women and 24 men), aged 55 to 65 years, to resveratrol supplementation or energy restriction for 30 days. Blood lipids, glucose, insulin, C-reactive protein, noradrenaline, SIRT1 (circulating and gene expression), and flow-mediated vasodilation (FMD) and nitrate-mediated vasodilation (NMD) were measured. *Results*: Both interventions increased circulating SIRT1 (*p* < 0.001). Pre- and post-tests changes of plasma noradrenaline were significant for both groups (resveratrol: *p* = 0.037; energy restriction: *p* = 0.008). Baseline circulating SIRT1 was inversely correlated with noradrenaline (r = −0.508; *p* < 0.01), and post-treatment circulating SIRT1 was correlated with NMD (r = 0.433; *p* < 0.01). Circulating SIRT1 was a predictor of FMD in men (*p* = 0.045), but not in women. SIRT1 was an independent predictor of NMD (*p* = 0.026) only in the energy restriction group. *Conclusions*: Energy restriction and resveratrol increased circulating SIRT1 and reduced sympathetic activity similarly in healthy adults. SIRT1 was independently associated with NMD only in the energy restriction group.

## 1. Introduction

Endothelial dysfunction is associated with almost all risk factors for atherosclerosis [1]. The endothelium is a key regulator of vascular homeostasis, acting not only as a barrier but as an active regulator of vascular homeostasis [2]. The use of vascular reactivity parameters, such as flow-mediated dilation (FMD) and nitroglycerin-mediated vasodilation (NMD), non-invasive techniques for assessing endothelial function, has proven useful for predicting events in high-risk populations [3].

Endothelial dysfunction can be caused by oxidative stress, which decreases NO production, activation, and bioavailability. Flow-mediated vasodilation is an endothelium-dependent process that reflects the relaxation of an artery when exposed to increased shear stress during post-occlusive reactive hyperemia [4] and has been inversely associated with cardiovascular risk [5,6] and risk factors such as body mass index (BMI), smoking, and blood lipids [7]. Furthermore, it has been shown that low FMD was associated with impaired endothelium-dependent relaxation of coronary arteries in high-risk individuals [8].

Vascular reactivity is modulated by the activation of the sympathetic nervous system (SNS) through alpha_1_- and beta_2_-adrenoreceptor signaling, which induce vasoconstriction and vasodilation, respectively [9,10]. It has been reported that impaired beta-adrenergic signaling is found in conditions such as aging, heart failure, metabolic syndrome, hypertension, obesity, and diabetes, leading to a chronic vasoconstrictive state and increased systemic noradrenaline release [10,11]. Previous studies also reported increased circulating noradrenaline, a marker of SNS activity, in individuals with advanced age and cardiovascular diseases and its risk factors [12,13,14,15]. Therefore, pharmacological and non-pharmacological sympatholytic interventions are possible strategies with therapeutical potential for these conditions, and may also affect vascular function improvement [16].

Sirtuins are a class of proteins that regulate several cellular processes, such as genome integrity and mitochondrial function, and have been associated with longevity in animal studies [17]. Seven sirtuins (SIRT1–SIRT7) have been discovered in mammals [18]. Among these proteins, SIRT1, an NAD+-dependent protein deacetylase, has been extensively studied due to its critical role in anti-inflammatory and antioxidant signaling response [17]. Furthermore, SIRT1 has been associated with sympatholytic effects and improved cardiovascular function in experimental studies [19,20].

Dietary energy restriction is an activator of SIRT1 [21], which mediates the longevity-increasing effect in animals [22]. However, long-term adherence to energy restriction is challenging. Therefore, the therapeutic potential of SIRT1 activators has been studied. Several compounds and foods have demonstrated SIRT1 activation, but resveratrol is the most widely studied [23,24]. Resveratrol is a non-flavonoid polyphenolic compound derived from stilbene and is the main representative of this group of substances [25]. It is naturally found in foods such as berries, peanuts, and grapes and has been linked to improvements in several cardiometabolic parameters, including reductions in blood pressure, glucose homeostasis, and blood lipids [26]. One of the most relevant effects of resveratrol is the antioxidative effect, which restores mitochondrial function, redox balance, and anti-inflammatory state, resulting in improved nitric oxide (NO) production and bioavailability in the vascular endothelium, ameliorating its function [25].

Despite the supposed therapeutic effects of SIRT1 activation, there is a lack of clinical evidence regarding SIRT1 and health conditions and outcomes. Few clinical studies have associated SIRT1 activation with lower noradrenaline levels, blood lipids, HDL particle function, glucose, and weight reduction [21,27,28]. As far as is known, no study assessed the effects of SIRT1 activation on vascular function parameters in healthy patients.

Therefore, this study aims to evaluate the effects of SIRT1 activation by resveratrol supplementation and energy restriction on vascular reactivity in healthy adults.

## 2. Materials and Methods

### 2.1. Study Design and Participants

A randomized trial conducted on 48 individuals compared the effects of resveratrol supplementation (500 mg/day) and energy restriction (1000 kcal/day) for 30 days on SIRT1 and vascular reactivity parameters. The study participants were 24 postmenopausal women (01 years of natural amenorrhea) and 24 men aged 55 to 65, all without previous cardiovascular diseases. Participants were healthy volunteers without chronic non-communicable diseases, normal physical examination, and normal resting electrocardiogram. After a washout period of 15 days without using any medications or supplements, the participants were randomly assigned to either caloric restriction or resveratrol groups in a 1:1 ratio according to sex. Subsequently, participants underwent a standardized interview, blood sample collection, anthropometric assessment, blood pressure and heart rate measurement, and vascular reactivity test. Such procedures were repeated at the end of the study.

Exclusion criteria were: BMI ≥ 30 kg/m^2^; smokers; hypertension (use of anti-hypertensive medication or systolic and diastolic blood pressure ≥140 mmHg and ≥90 mmHg, respectively); dyslipidemia (use of lipid-lowering medication or serum triglyceride concentrations ≥1.7 mmol/L or total cholesterol ≥6.2 mmol/L); fasting glucose ≥6.10 mmol/L or use of hypoglycemic medication; hormone replacement therapy; premenopausal women. Other exclusion criteria were any previous self-reported history of, or treatment for, chronic renal failure (serum creatinine ≥176.8 μmol/L), liver failure, or endocrine, hematological, respiratory, or metabolically clinically significant findings.

The analyzed clinical variables were age, weight, body mass index (BMI), waist circumference, blood pressure, and heart rate. Biochemical parameters included serum concentrations of triglycerides, total cholesterol, high-density lipoprotein cholesterol (HDL-c), low-density lipoprotein cholesterol (LDL-c), apolipoprotein A–I (apoA–I), apolipoprotein B (apoB), lipoprotein (a) (Lp(a)), non-esterified fatty acids (NEFA), glucose, insulin, high-sensitivity C-reactive protein (hsCRP), noradrenaline (NA), and serum levels and gene expression of SIRT1. Vascular reactivity parameters included endothelium-dependent and endothelium-independent vasodilation.

**Figure 1:**
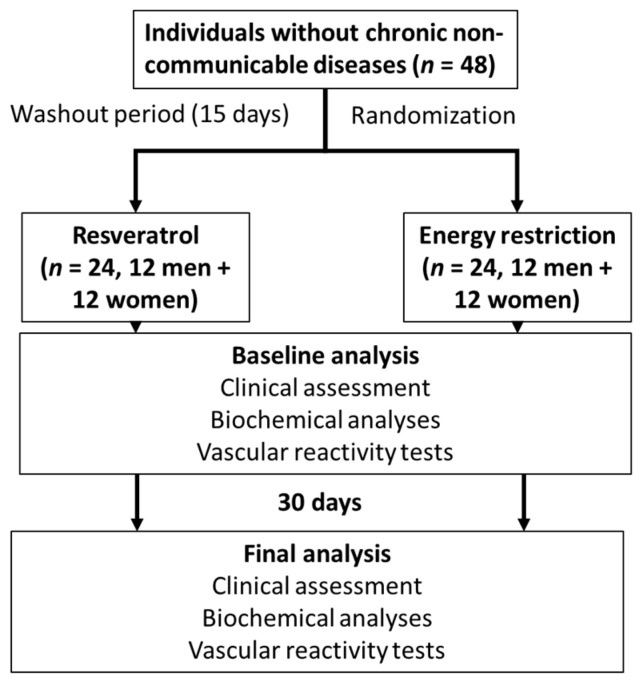
Research protocol design.

The energy restriction group consisted of a low-calorie diet (1000 kcal/day), corresponding to an approximate 50% energy intake reduction. Food diaries were used to analyze the adherence to the proposed interventions. Subjects were instructed to write down all the food intake daily and not exceed 1000 kcal/day. Participants included in the resveratrol group were instructed to take resveratrol capsules (250 mg of resveratrol each) twice a day (Figure 1).

The study was approved by the Ethics Committee of the University of São Paulo Medical School Hospital (CAAE:00788012.8.0000.0068), and all participants signed informed consent. Trial registration: www.clinicaltrials.gov; identifier: NCT01668836 (accessed on 27 June 2023).

### 2.2. Biochemical Analysis

Laboratory tests were performed with biological samples collected after a 12-h fast. Serum samples were obtained after collecting venous blood in tubes without an anticoagulant and centrifuged for 20 min at 1800G (Eppendorf, Hamburg, Germany). Citrated blood samples were centrifuged for 10 min at 200× *g* to obtain plasma rich in platelets (Eppendorf) and were used for aggregation tests. Serum total cholesterol, triglycerides, and HDL were obtained by commercial colorimetric-enzymatic methods (Cholesterol Oxidase Phenol Ampyrone-CHOD-PAP, Merck KGaA, Darmstadt, Germany). LDL was calculated using the Friedwald equation. Measurements were performed using a Dimension RxL (Siemens Healthcare Diagnostic Inc., Newark, DE, USA) with dedicated reagents. Lipoprotein (a), apoA-I, apoB, and hs-CRP determinations were made by immunone-phelometry using dedicated reagents for BN-II equipment from Siemens Healthcare (Marburg, Hessen, Germany). NEFA in serum was analyzed using a colorimetric kit from Randox Laboratories Ltd. (Crumlin, County Antrim, UK). Insulin was analyzed by chemiluminescence assay using automated equipment (Immulite 2000; Siemens Healthcare). All tests were performed according to the manufacturer’s instructions. Plasma NA was obtained through reversed-phase, ion-pair high-performance liquid chromatography (HPLC) coupled with electrochemical detection, following extraction by alumina adsorption according to a method previously described [29].

### 2.3. Sirtuin 1 Assessment

SIRT1 serum concentration was determined using an ELISA kit (Uscn Life Science, Wuhan, Hubei, China). Serum samples, before and after interventions, were analyzed in duplicate and in the same ELISA plate using the Multiscan FC plate reader (Thermo Scientific, Waltham, MA, USA), with a coefficient of variation of 12%, according to the manufacturer’s instructions. All tests were performed according to the manufacturer’s instructions.

Gene expression of SIRT1 was evaluated at pre- and post-inclusion in the protocol by using the specific assay Hs01009005_m1 (Applied Biosystems, Life Technologies, 151, Waltham, MA, USA). Total RNA was obtained from peripheral leukocytes using the TRIzol reagent (Life Technologies). cDNA synthesis was performed using a Superscript II kit (Life Technologies) with 1 μg from total RNA in a final volume of 20 μL, according to the manufacturer’s instructions. Expression of the housekeeping gene, glyceraldehyde 3-phosphate dehydrogenase (GAPDH), used as the normalizing gene, was evaluated by using the specific assay # Hs02758991_g1 (Applied Biosystems). The reaction mix was prepared using 5 μL of Universal Master Mix (Life Technologies), 0.5 μL of primers and probes mix (×20), and 2.5 μL of cDNA diluted samples (1:5).

PCR was performed according to the following protocol: enzymatic activation for 2 min at 50 °C, initial denaturation for 10 min at 95 °C, followed by 40 cycles of denaturation for 15 s at 95 °C and annealing for 20 s at 60 °C. Reactions were run in triplicate, and the number of copies of the gene transcript was determined using Ct (“threshold cycle”) values. For calculations, Ct values of SIRT1 were subtracted from Ct values for the GAPDH gene. The results are expressed in arbitrary units (AU).

### 2.4. Resveratrol Purity and Formulation Analysis

The resveratrol administered to participants was obtained from a compounding pharmacy (Buenos Aires Pharmacy, São Paulo, Brazil). The purity of the product supplied was analyzed by capillary electrophoresis using a Proteome Lab PA800 (Beckman Coulter, Fullerton, CA, USA) at the Laboratory of Capillary Chromatography and Electrophoresis at the Chemistry Institute of the University of São Paulo. Samples of the manipulated capsules and the standards of resveratrol were performed in triplicate, and areas under the peak were compared. The purity of resveratrol was 87 ± 1.1% on average (coefficient of variation: 1.2%).

### 2.5. Vascular Reactivity Assessment

Endothelium-dependent flow-mediated vasodilation (FMD) and endothelium-independent vasodilation (NMD) were assessed according to a previous guideline [30]. Brachial artery diameters were assessed in the left arm in the recumbent position after 10-min rest in a room kept at 20 °C to 25 °C, using a 7.5-MHz linear-array vascular ultrasound transducer and an Apogee 800 Plus ultrasound system (ATL Ultrasound, Bothell, WA, USA). Blood pressure and heart rate were monitored with an automated sphygmomanometer. Vessel diameter was measured in the longitudinal section, where the lumen-intima was viewed from the anterior to the posterior wall by software that measures a segment of the artery and calculates an average. Reactive hyperemia was induced by the inflation of a tourniquet around the forearm to 250 mm Hg and deflated after 5 min. After resting for 10 min, NMD was performed using sublingual isosorbide dinitrate at a dose of 5 mg, and the measurements of peak vasodilation were continuously recorded 3 min later. Endothelium-dependent and independent vasodilation were calculated as the percentage change in brachial artery diameter ratio after reactive hyperemia or isosorbide dinitrate to baseline diameter. All tests were performed and analyzed by a single dedicated ultra-sonographer according to recommendations of the International Brachial Artery Reactivity Task Force for endothelial function studies [30].

### 2.6. Statistical Analyses

The sample size of 48 patients, with 24 subjects per treatment arm, was determined to yield a power of 80% with a 5% significance level to detect a 30% difference in Sirt1 serum concentrations. Participants were randomly assigned in a 1:1 ratio using computer-generated numbers to include participants in the resveratrol or energy restriction groups.

Pre- and post-intervention variables were described as the median and interquartile range (IQR). Wilcoxon’s test was used for pre- and post-treatment analysis. Mann-Whitney’s U test was used for intergroup comparisons of baseline variables and changes. Partial correlations controlled by intervention group (resveratrol and energy restriction) and sex (men and women) were used to evaluate the relationship between changes (Δ) of SIRT1 and vascular reactivity. Multiple linear regressions with a backward method were used to assess this association further, using changes (Δ) of triglycerides, BMI, total cholesterol, and noradrenaline as adjustment variables and changes (Δ) of FMD and NMD as dependent variables. Predictive variables with weak association (*p* value of F > 0.10) with the outcome were excluded from the model. The multiple regression model with the highest predictive power (assessed by F value and R^2^) was chosen to be the final model. All regression assumptions were fulfilled (i.e., no multicollinearity, homoscedasticity, normally distributed and independent errors, independence of the outcome variables, and linearity of the variables).

The level of significance was set at *p* < 0.05, and the software used for statistical analysis was SPSS version 20.

## 3. Results

Baseline and post-intervention data are described in Table 1. There were no statistically significant differences in any of the variables between the resveratrol and the energy restriction groups at baseline.

At the end of the study, individuals in the energy restriction group had a significant reduction in weight (*p* = 0.020), BMI (*p* = 0.011), waist circumference (*p* = 0.010), total cholesterol (*p* = 0.011), and LDL-c (*p* = 0.034), and an increase in HDL-c (*p* = 0.013) and apoA-I (*p* = 0.019). Individuals in the resveratrol group presented an increase in serum total cholesterol (*p* = 0.030) and apoB (*p* = 0.029). Both interventions reduced plasma noradrenaline (resveratrol group: *p* = 0.037; energy restriction group: *p* = 0.008).

When comparing groups, there was a statistically significant difference between the changes (∆) in weight (*p* = 0.031), BMI (*p* = 0.030), total cholesterol (*p* = 0.001), LDL-c (*p* = 0.012), triglycerides (*p* = 0.048), apoB (*p* = 0.004), and glucose (*p* = 0.032), showing that energy restriction was efficient in reducing these parameters, while resveratrol had no effect on, or even increased, some of these parameters (Table 2).

Both interventions increased serum SIRT1 (*p* < 0.001) without differences between changes (*p* = 0.452), showing that this increase was similar between groups. No statistical differences were detected in SIRT1 expression and vascular reactivity parameters at the end of the study.

Correlations adjusted by group and sex are described in Table 3. Baseline circulating SIRT1 was negatively correlated with post-treatment and change of noradrenaline. A change in circulating SIRT1 was also negatively correlated with baseline noradrenaline and positively associated with a change in noradrenaline, which positively correlated with a change in NMD. Furthermore, post-treatment circulating SIRT1 was positively correlated with post-treatment NMD. Baseline SIRT1 expression was positively correlated with a change in FMD. Post-treatment SIRT1 expression was negatively correlated with post-treatment artery diameter.

Multiple linear regressions are shown in Table 4 and Table 5. The analyses were done by dividing by sex and intervention group.

Regarding the impact of SIRT1 on FMD (Table 4), we found no statistically significant associations in the energy restriction and resveratrol groups. However, triglycerides (β = 0.098; *p* = 0.033) and BMI (β = −3.923; *p* = 0.035) were significant predictors of FMD in the energy restriction group. When analyzed separately by sex, SIRT1 expression was positively associated with FMD (β = 2.201; *p* = 0.045) in men. Total cholesterol (β = 0.362; *p* = 0.001) and BMI (β = −3.844; *p* = 0.047) were also predictors of FMD in this group. No statistically significant associations were found in the women group. Circulating SIRT1 was an independent predictor of NMD (β = 1.594; *p* = 0.026) in the energy restriction group (Table 5). We found no other significant predictor of NMD in the resveratrol, men, and women groups.

## 4. Discussion

The main results of our study showed that 30 days of energy restriction, i.e., a 1000 kcal diet, or 500 mg daily resveratrol supplementation similarly increased circulating SIRT1 and decreased plasma noradrenaline. Plasma noradrenaline reduction was correlated with higher baseline circulating SIRT1. After the interventions, circulating SIRT1 was positively correlated with NMD independently of sex and interventions. Furthermore, circulating SIRT1 was independently associated with NMD in the energy restriction group. Regarding SIRT1 gene expression, we did not find any statistically significant differences at the end of the study, but we found a statistically significant association with FMD in men.

Dietary energy restriction is the only physiological intervention that increases life expectancy in mammals [31]. It is also an important clinical nutrition intervention used to treat excess body fat and its cardiometabolic comorbidities, improving biomarkers such as blood glucose, insulin resistance, triglycerides, LDL-c, systolic blood pressure, and hs-CRP [32]. The benefits of these interventions follow a dose-dependent pattern. However, long-term adherence remains an issue, as evidenced in the CALERIE study, which showed a significant loss of adherence after six months of intervention with moderate energy restriction [32,33]. One of the mechanisms by which energy restriction mediates its beneficial cardiometabolic effects is due to the activation of SIRT1 [21]. The mechanism behind SIRT1 activation by energy restriction is the decline of glycolytic rates in favor of respiratory metabolism as the main energy source, leading to nicotinamide adenine dinucleotide (NAD^+^) replenishment and reduction of NADH, a competitive inhibitor of SIRT1 deacetylase activity, leading to SIRT1 activation. Energy restriction also increases pyrazine-amidase and nicotine-amidase 1 (PNC1) and nicotinamide phosphoribosyl-transferase (Nampt) expression, inducing NAD^+^ resynthesis from nicotinamide (NAM) [22].

Resveratrol increases cell cAMP content, which in turn activates monophosphate-activated protein kinase (AMPK), a protein that acts as an upstream molecule to regulate expression of SIRT1 [34]. Our study showed that 500 mg resveratrol supplementation increased circulating SIRT1 in a manner equivalent to a diet of severe energy restriction, being a potential energy restriction mimetic. However, our results differ from a study that compared the effects of 600 mg of resveratrol and a low-calorie diet in patients with non-alcoholic fatty liver disease (NAFLD) [35]. Other studies showed that resveratrol is ineffective in patients with liver diseases and obesity, which alters resveratrol kinetics, explaining the divergence from the results of our study regarding serum SIRT1 [36,37]. Another possible explanation is that high doses of resveratrol may have resulted in saturation in absorption sites, limiting its therapeutic effect. It is important to note that these studies used doses three to six times higher than ours [36,37].

SIRT1 has gained attention due to its metabolic regulation effect with concomitant activation of AMPK (adenosine5′-monophosphate (AMP)-activated protein kinase), a key molecule in cellular metabolism. Both proteins induce mitochondrial biogenesis, fatty acids β-oxidation, and gluconeogenesis through the activation of peroxisome proliferator-activated receptor- γ coactivator 1α (PGC1α) [38]. By improving mitochondrial function, the induction of PGC1α by SIRT1 also reduces oxidative stress caused by mitochondrial disorders. Furthermore, SIRT1 promotes the expression of mitochondrial superoxide dismutase (SOD2) and catalase by activating fork-head box protein O3a (FOXO3a), increasing cellular antioxidant capacity [39]. Moreover, SIRT1 inhibits inflammation by direct deacetylation of the nuclear factor-κB (NF-κB) p65 subunit, thereby repressing transcription of NF-κB genes [40,41].

Inflammation and oxidative stress are key factors for nitric oxide (NO) degradation and, subsequently, endothelial dysfunction, which is a critical factor for the development and progression of cardiovascular diseases [42]. Flow-mediated vasodilation is a non-invasive method that has been used for the diagnosis of vascular dysfunction in high-cardiovascular-risk individuals. However, there are still issues regarding cut-off values and applicability in low-risk individuals [43]. One study proposed a cut-off value of 7.1% of FMD and 15.6% of NMD for individuals without risk factors or established cardiovascular disease [44]. Compared to this cut-off value, our study subjects presented lower FMD and NMD, showing that endothelial dysfunction was present in our sample. This result can be explained by our study’s slightly increased BMI, waist circumference, and total cholesterol. These risk factors were associated with endothelial dysfunction in a large population-based cohort focused on the analysis of endothelial cell-derived proteins, showing that changes in vascular function can be detected early in individuals with risk factors [45].

Our results showed that baseline SIRT1 gene expression positively correlated with changes in FMD, suggesting that individuals with higher SIRT1 gene expression presented higher FMD. Furthermore, we showed that SIRT1 expression was positively associated with FMD in men. This may be explained by the lower levels of circulating estrogen in men than in women, resulting in lower basal activation of SIRT1 in this group. Laboratory studies showed that, in female rats, estrogen activated the SIRT1/AMPK signaling pathway, increasing protection against cerebral ischemic stress [46] and angiotensin (Ang) II-induced cardiomyocyte hypertrophy [47], which could explain the mechanisms behind the epidemiological differences of cardiovascular diseases between men and women [48,49]. Previous studies showed differences between men and women regarding sirtuin levels in heart and skeletal muscle tissues [50,51], corroborating the hypothesis that women have an increased baseline sirtuin content, probably due to higher levels of circulating estrogens. Therefore, our results support that interventions that increase SIRT1 may be more effective in men with lower baseline SIRT1. Although our results showed that circulating SIRT1 increased after both interventions, it was not significantly associated with FMD. However, BMI, which is an important predictor of circulating SIRT1 in previous studies [21,52], was a significant predictor of FMD in men. Although questions have been raised about using FMD for cardiovascular event prediction in low-risk populations [43], a meta-analysis showed that it is a useful prognostic marker in these individuals [53]. Our results suggest that energy restriction may improve FMD in men through the increase of circulating SIRT1 and BMI reduction. However, the interpretation of our results should be done carefully, since only gene expression of SIRT1, and not circulating levels, was associated with FMD in multiple regressions. It is important to note that we did not find significant differences of SIRT1 expression or FMD after energy restriction (Table 1), but only correlations (baseline SIRT1 expression and ΔFMD) which may not necessarily reflect causality. Women did not have this association because, perhaps, of the fact that they presented, in the basal state, higher SIRT1 [50,51] and FMD [54] than men due to estrogens, and the interventions of the present study were not sufficient to optimize these parameters in this group.

Regarding endothelium-independent vasodilation, our results showed that circulating SIRT1 was independently associated with NMD in the energy restriction group. Vascular reactivity measured by NMD provides information about the response of vascular smooth muscle cells (VSMCs) to a NO donor, i.e., endothelium-independent vasodilation [55]. It has been demonstrated that patients suffering from coronary heart disease presented blunted vasodilator responses to increased blood flow and intracoronary injection of nitroglycerin, showing that impaired NMD may be associated with structural vascular alterations involving VSMCs as a result of atherosclerotic lesions, expressing a more advanced state of the disease [56]. Other studies have found associations between NMD and blunted nocturnal blood pressure fall in hypertensive patients [57], the presence and quantity of coronary artery calcium in asymptomatic patients [58], and albuminuria [59]. In a prospective study, NMD was an independent predictor of long-term cardiovascular events in subjects with and without evidence of atherosclerotic diseases [60]. Furthermore, it was found that NMD decreased with cumulative cardiovascular risk factors and Framingham cardiovascular risk score. The authors argue that NMD reflects risk better than FMD, since the assessment of FMD is based on the premise that endothelium-independent vasodilation is not altered, and a significant difference in NMD was found between individuals with and without cardiovascular diseases [61]. Therefore, the independent association of circulating SIRT1 with NMD found in our study suggests that SIRT1 may decrease vascular remodeling that precedes cardiovascular disease, since alterations in NMD may reflect VSMC function. This corroborates previous studies that showed that SIRT1 activation suppressed factors involved in vascular remodeling, such as angiotensin II type I receptor (AT1R) expression [62], reduced DNA repair and apoptosis induction in VSMCs [63], hyperphosphatemia-induced arterial calcification [64], and vascular senescence and inflammation [65].

Both interventions reduced plasma noradrenaline, indicating a sympatholytic effect of resveratrol and dietary energy restriction. We also found that higher baseline levels of circulating SIRT1 were inversely correlated with plasma noradrenaline, suggesting that SIRT1 regulates plasma noradrenaline. Vascular reactivity is regulated by sympathetic nervous system (SNS) activation, which can be assessed by plasma noradrenaline [9,28]. Aging and age-related diseases have markedly increased plasma noradrenaline [11]. It was shown that SIRT1 activates the transcription of the gene encoding the monoamine oxidase A (MAO-A), an enzyme that plays a role in the metabolization of neurotransmitters. It was found that brain-specific SIRT1 knockout mice had higher levels of noradrenaline [20]. Increased plasma noradrenaline and sympathetic vasomotor activity associated with augmented protein expression of AT1R and attenuated SIRT1 protein expression were also found in the offspring of rats exposed to a high-fructose diet [66]. This evidence shows that SIRT1 plays a role in the regulation of noradrenaline. Our findings support that SIRT1 activation may have a sympatholytic effect, since a reduction in noradrenaline was observed. However, correlations between circulating SIRT1 were not maintained after treatment. This was an unexpected result and the reason behind this could be that, probably, not all participants responded similarly, since the reduction in NA in the resveratrol group was less than in the energy restriction group, despite not significantly (Table 2). Furthermore, the responses to the different treatments may have affected the sympatholytic effects observed. This implies the need for caution in interpreting and generalizing our results, and the need for future studies on how sirtuin affects noradrenaline kinetics.

Our study has limitations. Firstly, we analyzed a small number of subjects. Our sample size was calculated using circulating SIRT1. Therefore, variations in parameters such as gene expression of SIRT1 and vascular reactivity could be undetected. On the other hand, we used multivariate analysis and correlations to detect change tendencies. Another limitation was that all subjects were at low cardiovascular risk, which could mask alterations in vascular reactivity parameters after interventions. In addition, the applicability of vascular reactivity tests on low cardiovascular risk populations is still debated [43]. Furthermore, few studies have shown that resveratrol supplementations did not change biomarkers in healthy subjects [67]. The limitations concerning the use of plasma NA were: (I) only a small fraction of NA diffused into plasma where it was measured; (II) plasma NA concentration was dependent on clearance rate, and not only sympathetic tonus and NA secretion; and (III) the sources of plasma NA were not identified, though regional sympathetic responses cannot be measured [68].

As far as we know, this is the first study that associated SIRT1 with well-established markers of vascular function in humans. Our results corroborate a previous laboratory study that showed that SIRT1 improves endothelial function [69]. Further studies should focus on how SIRT1 is altered in cardiometabolic diseases and if stimulation of SIRT1 by resveratrol could ameliorate vascular functions in these populations.

## 5. Conclusions

Our study showed that 30 days of energy restriction (1000 kcal diet) or 500 mg daily resveratrol increased circulating SIRT1 and decreased plasma noradrenaline similarly. However, circulating SIRT1 was independently associated with NMD only in the energy restriction group, showing that mechanisms other than the increase in circulating SIRT1 present in calorie restriction, and that resveratrol was unable to activate, may be responsible for cardiovascular protection.

In addition, energy restriction and resveratrol did not change SIRT1 gene expression in leukocytes, but the expression was positively associated with FMD in men after multivariate analysis. It is difficult to draw conclusions about the use of gene expression in leukocytes for assessment of overall SIRT1, since circulating SIRT1 increased, and its influence on endothelial function measured by FMD. Therefore, our study showed that leukocyte SIRT1 gene expression does not reflect circulating SIRT1, and futures studies should rely on more biomarkers of SIRT1 biochemical pathways to achieve a better understanding of the role of this protein in vascular function and metabolism.

## Figures and Tables

**Table 1:** Clinical and biochemical characteristics of the participants at baseline and post-treatment.

Variables	Resveratrol					Energy Restriction					
	Baseline		Post-Treatment		*p* *	Baseline		Post-Treatment		*p* *	Baseline *p* **
	*n* = 24		*n* = 24			*n* = 24		*n* = 24			
	Median	(IQR)	Median	(IQR)		Median	(IQR)	Median	(IQR)		
*Clinical characteristics*											
Age, y	58.0 (56.0–61.0)				N/A	59.0 (55.25–60.75)				N/A	0.764
Women, n	12 (50%)				N/A	12 (50%)				N/A	N/A
Weight, kg	78.4	(63.4–91.8)	79.9	(63.0–92.4)	0.569	71.0	(62.0–83.5)	70.4	(60.5–81.3)	**0.020**	0.210
Body mass index, kg/m^2^	26.5	(24.8–30.7)	26.7	(24.8–31.1)	0.587	25.9	(23.4–27.9)	25.5	(23.0–27.6)	**0.011**	0.165
Waist circumference, cm	93.5	(87.5–105.0)	94.0	(88.5–107.3)	0.793	92.5	(88.8–100.3)	91.5	(84.8–97.3)	**0.010**	0.664
Heart rate, bpm	64.0	(59.0–71.0)	65.5	(61.0–70.0)	0.379	62.5	(54.0–71.0)	64.0	(53.3–70.0)	0.968	0.674
SBP, mmHg	130.5	(122.0–143.5)	133.0	(113.5–142.0)	0.681	132.5	(112.8–138.3)	122.0	(117.0–131.8)	0.188	0.991
DBP, mmHg	80.0	(73.8–86.5)	83.0	(74.5–89.0)	0.614	83.0	(72.8–92.0)	77.5	(72.0–84.3)	0.091	0.700
*Biochemical characteristics*											
Total cholesterol, mg/dL	202.0	(185.5–226.8)	215.5	(177.8–245.3)	**0.030**	211.0	(190.8–245.3)	201.0	(176.3–231.0)	**0.011**	0.421
HDL–c, mg/dL	48.0	(38.8–57.0)	46.5	(37.3–57.0)	0.329	50.5	(42.5–63.8)	52.0	(39.8–59.5)	**0.013**	0.293
LDL–c, mg/dL	130.5	(111.5–149.5)	149.0	(98.3–165.3)	0.095	143.5	(108.0–158.8)	133.0	(99.3–160.5)	**0.034**	0.433
Triglycerides, mg/dL	110.0	(68.8–177.8)	128.0	(81.5–186.0)	0.170	94.5	(72.5–128.0)	82.0	(56.8–116.8)	0.074	0.557
Apoliproprotein A–I, g/L	1.49	(1.29–1.62)	1.37	(1.25–1.62)	0.678	1.45	(1.35–1.73)	1.38	(1.23–1.57)	**0.019**	0.523
Apolipoprotein B, g/L	0.92	(0.83–1.10)	1.00	(0.92–1.17)	**0.029**	0.99	(0.79–1.21)	0.96	(0.74–1.08)	0.052	0.657
Lp(a), mg/dL	10.9	(3.2–25.1)	9.7	(2.8–21.6)	0.362	12.0	(5.4–37.4)	14.6	(6.7–35.1)	0.498	0.523
Glucose, mg/dL	93.0	(84.3–108.0)	96.0	(87.3–107.0)	0.201	92.5	(86.0–99.0)	90.0	(84.8–93.8)	0.187	0.749
Insulin, µUI/mL	6.4	(5.0–10.6)	7.8	(5.3–9.1)	0.378	5.0	(3.6–8.3)	5.7	(2.8–8.3)	0.513	0.180
NEFA, mEq/dL	0.21	(0.15–0.34)	0.25	(0.16–0.30)	0.909	0.20	(0.09–0.42)	0.19	(0.04–0.29)	0.330	0.676
hs–CRP, mg/L	1.81	(0.81–3.00)	1.46	(0.70–2.81)	0.904	1.36	(0.79–2.27)	1.18	(0.77–2.21)	0.131	0.645
Noradrenaline, pg/dL	256.0	(183.8–305.5)	228.5	(148.3–267.5)	**0.037**	322.5	(220.0–434.0)	190.0	(117.0–414.8)	**0.008**	0.078
*Sirtuin 1*											
Circulating sirtuin 1, ng/mL	0.78	(0.78–0.92)	6.90	(3.03–7.79)	**<0.001**	0.78	(0.78–1.88)	6.13	(4.21–7.63)	**<0.001**	0.349
Sirtuin 1 expression, AU	10.70	(9.80–12.40)	11.40	(10.68–12.22)	0.931	11.13	(10.17–12.09)	10.76	(8.98–12.42)	0.289	0.981
*Vascular reactivity*											
Artery diameter, mm	4.20	(3.90–4.70)	4.00	(3.70–5.00)	0.943	4.50	(3.70–5.30)	4.30	(3.70–5.40)	0.682	0.729
FMD, %	2.38	(0.00–4.87)	2.49	(0.00–6.00)	0.758	3.88	(0.00–5.31)	1.97	(0.00–6.10)	0.443	0.538
NMD, %	14.20	(11.40–23.80)	16.20	(12.50–22.80)	0.959	14.35	(12.50–21.35)	17.60	(12.15–26.98)	0.196	1.000

Data are presented as median (interquartile range—IQR). *: Comparison of intra-groups post–pre-tests. **: Comparison between groups at baseline. Significant values are bold.

**Table 2:** Comparison of the median differences (post-test—pre-test; ∆) between groups.

Variables	Resveratrol (*n* = 24)		Energy Restriction (*n* = 24)		Difference (∆) *p*
	Median Difference (∆)	(IQR)	Median Difference (∆)	(IQR)	
*Clinical characteristics*					
Weight, kg	0	(−0.7 to +1.4)	−1.4	(−2.4 to +0.5)	**0.031**
Body mass index, kg/m^2^	0	(−0.2 to +0.4)	−0.4	(−0.7 to 0.0)	**0.030**
Waist circumference, cm	−0.5	(−3.0 to +2.0)	−1	(−4.0 to +0.3)	0.223
Heart rate, bpm	0	(−3.0 to +5.0)	0	(−4.0 to +4.0)	0.582
Systolic blood pressure, mmHg	0	(−11.0 to +9.5)	−0.5	(−20.0 to +8.0)	0.375
Dyastolic blood pressure, mmHg	+1.0	(−4.5 to +5.5)	−2.0	(−6.3 to +1.3)	0.141
*Biochemical characteristics*					
Total cholesterol, mg/dL	+11.5	(−8.8 to +27.5)	−13.0	(−24.5 to +3.0)	**0.001**
HDL-c, mg/dL	−1.0	(−4.0 to 2.8)	−3.5	(−8.0 to 0.0)	0.082
LDL-c, mg/dL	+10.5	(−10.3 to +24.0)	−8.0	(−21.8 to +6.8)	**0.012**
Triglycerides, mg/dL	+11.5	(−19.8 to +49.5)	−14.5	(−27.5 to +5.5)	**0.048**
Apoliproprotein A–I, g/L	−0.03	(−0.12 to +0.10)	−0.07	(−0.25 to +0.02)	0.108
Apolipoprotein B, g/L	+0.09	(−0.05 to +0.14)	−0.08	(−0.17 to +0.4)	**0.004**
Lp(a), mg/dL	−0.1	(−2.3 to +0.6)	0.0	(−1.5 to +0.7)	0.813
Glucose, mg/dL	+4.0	(−4.3 to +8.5)	−1.5	(−12.0 to +4.8)	**0.032**
Insulin, µUI/mL	−0.3	(−1.0 to +1.9)	−0.3	(−2.3 to +2.1)	0.366
NEFA, mEq/dL	−0.03	(−0.09 to 1.00)	−0.01	(−0.16 to 0.06)	0.509
hs-CRP, mg/L	−0.02	(−0.88 to +0.41)	−0.23	(−0.92 to +0.15)	0.394
Noradrenaline, pg/dL	−27.0	(−96.3 to +9.5)	−74.5	(−198.3 to −12.5)	0.138
*Sirtuin-1*					
Circulating Sirtuin-1, ng/mL	+5.9	(+2.0 to +7.0)	+4.1	(+1.9 to +6.4)	0.452
Sirtuin-1 expression, AU	+0.63	(−1.91 to +1.18)	−0.71	(−1.46 to +0.88)	0.95
*Vascular reactivity*					
Baseline artery diameter, mm	0.00	(−0.20 to +0.20)	+0.05	(−0.15 to +0.20)	0.663
FMD, %	0.00	(−4.25 to +5.33)	−0.97	(−5.35 to +3.14)	0.499
NMD, %	0.00	(−7.10 to +2.10)	+1.15	(−2.40 to +7.60)	0.313

Data are presented as median (interquartile range—IQR). Significant values are bold.

**Table 3:** Partial correlations between vascular reactivity parameters, noradrenaline, and Sirtuin 1 adjusted by treatment group and sex.

Variables	Baseline				Post-Treatment				Post- Minus Pre-Treatment Change (Δ)			
	AD	FMD	NMD	NA	AD	FMD	NMD	NA	AD	FMD	NMD	NA
*Baseline*												
Circulating sirtuin 1	−0.146	0.107	0.322	0.198	0.029	−0.023	0.234	−0.310 *	0.208	−0.096	−0.061	−0.508 **
Sirtuin 1 expression	−0.038	−0.247	−0.314	0.001	−0.158	−0.280	−0.035	0.022	−0.253	0.368 *	0.239	0.021
Noradrenaline	−0.203	−0.202	0.174	1.000	−0.201	−0.320	0.041	0.506 **	−0.018	−0.037	−0.115	−0.526 **
*Post-treatment*												
Circulating sirtuin 1	−0.187	0.177	0.259	0.187	−0.094	−0.081	0.433 **	−0.228	0.159	−0.185	0.187	−0.117
Sirtuin 1 expression	−0.259	−0.001	−0.115	−0.017	−0.365 *	0.164	0.129	0.014	−0.230	0.097	0.234	0.032
Noradrenaline	−0.235	−0.167	−0.277	0.506 **	−0.336 *	−0.069	0.045	1.000	−0.218	0.087	0.290	0.467 **
*Change* (Δ)												
Circulating sirtuin 1	−0.088	0.101	0.063	−0.299 *	−0.066	−0.059	0.260	0.041	0.033	−0.114	0.195	0.347 *
Sirtuin 1 expression	−0.342	0.245	0.201	−0.013	−0.292	−0.049	0.151	−0.033	0.036	−0.222	−0.038	−0.021
Noradrenaline	−0.020	0.042	−0.434 **	−0.526 **	−0.118	0.250	0.004	0.467 **	−0.188	0.119	0.390 *	1.000

Values are detailed as correlation coefficient (r). AD: artery diameter; FMD: flow-mediated vasodilation; NA: noradrenaline; NMD: nitrate-mediated vasodilation; *: *p* < 0.05; **: *p* < 0.01. Significant values are bold.

**Table 4:** Effects of Sirtuin 1 on flow-mediated vasodilation stratified by sex and intervention.

Predictor Variables	Final Model				
	R^2^	β	95% CI for β		*p*
			Lower	Upper	
*Resveratrol group*	0.472				
Constant		1.145	−10.787	13.077	0.837
Triglycerides		n/a	n/a	n/a	n/a
BMI		3.850	−3.881	11.580	0.296
Total cholesterol		n/a	n/a	n/a	n/a
Noradrenaline		n/a	n/a	n/a	n/a
Circulating sirtuin 1		−0.448	−2.539	1.642	0.646
Sirtuin 1 expression		−2.374	−6.031	1.282	0.181
*Energy restriction group*	0.383				
Constant		−1.880	−5.207	1.447	0.246
Triglycerides		0.098	0.009	0.188	**0.033**
BMI		−3.923	−7.534	−0.311	**0.035**
Total cholesterol		n/a	n/a	n/a	n/a
Noradrenaline		n/a	n/a	n/a	n/a
Circulating sirtuin 1		n/a	n/a	n/a	n/a
Sirtuin 1 expression		n/a	n/a	n/a	n/a
*Men*	0.774				
Constant		4.844	−1.487	11.175	0.122
Triglycerides		n/a	n/a	n/a	n/a
BMI		−3.844	−7.637	−0.052	**0.047**
Total cholesterol		0.362	0.176	0.549	**0.001**
Noradrenaline		n/a	n/a	n/a	n/a
Circulating sirtuin 1		−1.235	−2.507	0.038	0.056
Sirtuin 1 expression		2.201	0.054	4.349	**0.045**
*Women*	0.476				
Constant		−1.026	−7.575	5.523	0.734
Triglycerides		n/a	n/a	n/a	n/a
BMI		n/a	n/a	n/a	n/a
Total cholesterol		n/a	n/a	n/a	n/a
Noradrenaline		n/a	n/a	n/a	n/a
Circulating sirtuin 1		n/a	n/a	n/a	n/a
Sirtuin 1 expression		−3.123	−7.184	0.938	0.117

The first model included all variables (i.e., triglycerides, BMI, total cholesterol, noradrenaline, circulating, and expression of Sirtuin 1). Excluded variables in the final model are indicated by “n/a”. Significant values are bold.

**Table 5:** Effects of Sirtuin 1 on nitrate-mediated vasodilation stratified by sex and intervention.

Variables	Final Model				
	R^2^	β	95% CI for β		*p*
			Lower	Upper	
*Resveratrol group*	0.570				
Constant		−2.118	−6.719	2.483	0.336
Triglycerides		0.063	−0.006	0.132	0.071
BMI		n/a	n/a	n/a	n/a
Total cholesterol		n/a	n/a	n/a	n/a
Noradrenaline		n/a	n/a	n/a	n/a
Circulating sirtuin 1		n/a	n/a	n/a	n/a
Sirtuin 1 expression		−1.231	−3.624	1.163	0.285
*Energy restriction group*	0.538				
Constant		−3.874	−10.551	2.802	0.235
Triglycerides		n/a	n/a	n/a	n/a
BMI		n/a	n/a	n/a	n/a
Total cholesterol		n/a	n/a	n/a	n/a
Noradrenaline		n/a	n/a	n/a	n/a
Circulating sirtuin 1		1.594	0.221	2.966	**0.026**
Sirtuin 1 expression		n/a	n/a	n/a	n/a
*Men*	0.454				
Constant		1.624	−8.279	11.526	0.730
Triglycerides		n/a	n/a	n/a	n/a
BMI		n/a	n/a	n/a	n/a
Total cholesterol		n/a	n/a	n/a	n/a
Noradrenaline		0.023	−0.012	0.058	0.183
Circulating sirtuin 1		0.261	−1.512	2.044	0.758
Sirtuin 1 expression		−0.820	−3.524	1.885	0.526
*Women*	0.363	n/a	n/a	n/a	n/a
Constant		−1.517	−13.670	10.636	0.781
Triglycerides		0.046	−0.057	0.149	0.331
BMI		n/a	n/a	n/a	n/a
Total cholesterol		n/a	n/a	n/a	n/a
Noradrenaline		n/a	n/a	n/a	n/a
Circulating sirtuin 1		0.427	−1.739	2.594	0.661
Sirtuin 1 expression		0.081	−3.959	4.121	0.964

The first model included all variables (i.e., triglycerides, BMI, total cholesterol, noradrenaline, circulating, and expression of Sirtuin 1). Excluded variables in the final model are indicated by “n/a”. Significant values are bold.

## Data Availability

Not applicable.
